# Comparative Effects of Facial Roller and Gua Sha Massage on Facial Contour, Muscle Tone, and Skin Elasticity: Randomized Controlled Trial

**DOI:** 10.1111/jocd.70236

**Published:** 2025-05-29

**Authors:** Sun‐hee Ahn, Ui‐jae Hwang, Hyo sun Han, Jun‐hee Kim, Hyun‐joo Lee, Yu‐rin Jeon, Hyun Hwa Lee, A‐hyun Hwang

**Affiliations:** ^1^ Department of Physical Therapy, College of Software and Digital Healthcare Convergence Yonsei University Wonju‐si Gangwon‐do Republic of Korea; ^2^ Department of Rehabilitation Sciences The Hong Kong Polytechnic University Hong Kong SAR China; ^3^ Department of Beauty Design Bucheon University Bucheon Republic of Korea; ^4^ Department of Pharmacy College of Pharmacy, Dankook University Cheonan‐si Chungcheongnam‐do Republic of Korea; ^5^ Department of Information & Statistics Korea University Sejong‐si Republic of Korea

**Keywords:** face lift, nasolabial fold, rejuvenation, skin elasticity

## Abstract

**Background:**

This study aimed to compare the effects of facial roller and gua sha massage on anthropometric facial contours, muscle tone, and skin elasticity parameters during an 8‐week intervention period.

**Methods:**

Thirty‐four women aged 20–50 years were randomly assigned to facial roller (*n* = 17) or gua sha (*n* = 17) groups. Participants performed the designated massage technique for 10 min, five times per week for 8 weeks. Outcome measures included facial surface distances (subnasale‐to‐sublobular distance, mid‐point distance, labrale superius distance, jawline surface distance), muscle tone parameters (oscillation frequency [*F*], dynamic stiffness [s]), and skin elasticity indices (gross elasticity [R2], biological elasticity [R7]).

**Results:**

Both groups showed significant improvements in facial contour measurements, with reductions ranging from 2.23 to 2.40 mm in the gua sha group (*p* < 0.001 for all measurements) and 2.75–3.26 mm in the facial roller group (*p* < 0.001 for all measurements). The gua sha group demonstrated significant reductions in muscle tone parameters (*F*: −2.02 Hz, *p* < 0.001; *S*: −56.46 N/m, *p* = 0.002), while the facial roller group showed significant improvements in skin elasticity (R2: 8.6%, R7: 7.5%, *p* < 0.001). The between‐group differences were significant for both muscle tone (*p* < 0.05) and skin elasticity parameters (*p* < 0.05).

**Conclusions:**

Both interventions effectively improved facial contours through distinct physiological mechanisms: gua sha primarily through changes in muscle properties and facial roller through enhanced skin elasticity. These findings support targeted treatment selection based on specific therapeutic goals in facial aesthetic practice.

## 1 Introduction

Facial massage has been a cornerstone of traditional therapeutic and cosmetic practices since its first mention in the ancient Chinese medical text “Huangdi Neijing” around 2700 bc [1, 2]. In recent years, facial massage techniques have evolved to incorporate various tools and methods, with facial rollers and gua sha becoming increasingly popular for their purported benefits of facial contouring and skin health enhancement [3].

Recent studies have demonstrated that facial roller massage significantly affects the skin's blood flow and vascular reactivity. Miyaji et al. [4] found that 5 min of roller massage increased skin blood flow for at least 10 min post‐treatment, promoting lymphatic drainage and potentially improving the skin condition. This enhanced blood circulation may directly influence skin elasticity by improving the delivery of oxygen and nutrients to skin cells while promoting the removal of metabolic waste products [5]. Mechanical stimulation provided by facial rollers has been shown to promote lymphatic drainage and enhance blood circulation through gentle rolling motions [6]. These circulatory improvements may contribute to better skin elasticity and texture by supporting optimal skin cell function and mechanotransduction pathways that regulate tissue architecture and stability [5].

In contrast, gua sha massage, as a form of Instrument Assisted Soft Tissue Mobilization (IASTM), involves scraping the skin with a smooth‐edged tool, affecting both superficial and deeper tissue layers. Nielsen et al. [1] demonstrated that gua sha treatment significantly increased microcirculation in surface tissue and promoted therapeutic effects on deeper fascia. This technique increases local microperfusion by up to 400% and maintains increased circulation for over 25 min after treatment. The mechanical stimulus provided by IASTM techniques such as gua sha has been shown to influence both the extracellular matrix and cellular responses of muscle and fascial tissues [7]. Cheatham et al. [8] reported that IASTM techniques effectively reduce tissue restriction and muscle tension by targeting facial adhesions and promoting the remodeling of soft tissue structures. This mechanical loading of facial tissues during gua sha treatment may stimulate fibroblast activity and collagen synthesis, potentially leading to improved tissue organization and reduced muscle tone [9].

Despite the widespread use of both facial rollers and gua sha in aesthetic practice, there are several critical gaps in the current literature. While previous studies have documented the individual effects of these techniques on skin blood flow [4] and tissue microcirculation [1], there is limited comparative research examining their differential effects on facial anthropometric measurements, muscle tone, and skin elasticity over an extended period. Furthermore, although these techniques are commonly used for facial contouring, quantitative evidence comparing their effectiveness using standardized measurements is lacking [6]. The need for this research is particularly pressing given the growing demand for evidence‐based, non‐invasive facial contouring techniques in both clinical and aesthetic settings [3].

The distinction in mechanical principles and depth of tissue engagement between these two techniques suggests potentially different outcomes in facial contouring. While facial rollers primarily affect superficial circulation and lymphatic drainage [4], gua sha's deeper tissue manipulation through IASTM mechanics may lead to more substantial changes in facial muscle tone and fascial organization [8]. Understanding these differential effects is crucial for practitioners to make evidence‐based recommendations for specific facial contouring goals and to optimize treatment outcomes for different patient needs.

Therefore, this study aims to compare the effects of facial roller and gua sha massage on facial contours, muscle tone, and skin elasticity over an eight‐week intervention period. Specifically, we will evaluate changes in anthropometric facial measurements, tissue properties, and skin elasticity parameters using three‐dimensional scanning and biomechanical assessment tools. We hypothesize that: (1) both interventions will significantly improve facial contour measurements compared to baseline; (2) gua sha massage will show greater improvements in muscle tone and deeper tissue‐related parameters; and (3) facial roller massage will demonstrate greater improvements in skin elasticity.

## 2 Methods

### 2.1 Subjects

**FIGURE 1:**
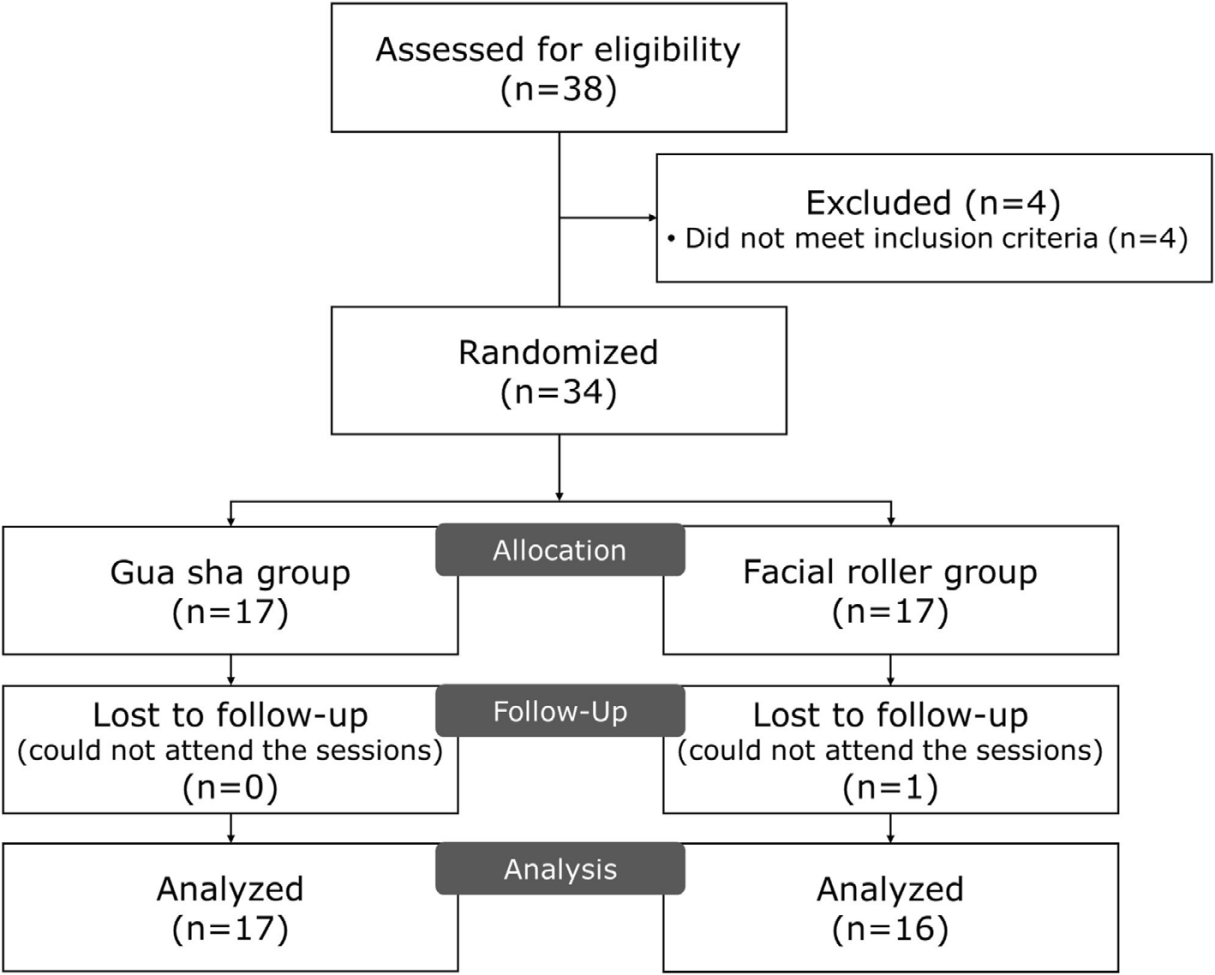
Flow diagram of our randomized trial of Gua sha and facial roller massage.

A total of 34 women aged between 20 and 50 years were recruited from Seoul and Gyeonggi‐do regions between April and July 2024. The study protocol was reviewed and approved by the Institutional Review Board of Yonsei University Mirae (approval number: 1041849‐202 401‐BM‐017‐03). All measurements were conducted in a controlled laboratory setting, where temperature and humidity were maintained at 22°C–24°C and 40%–60%, respectively. The sample size calculation was performed using G*Power 3.1 software (Heinrich Heine University Dusseldorf, Germany), which indicated that 30 participants were required to detect significant differences with an effect size of 0.274, a significance level (α) of 0.05, and a power (1‐β) of 0.8 in a priori power analysis. While our initial sample size calculation was based on a conservative effect size estimate of 0.274 in a priori power analysis, post hoc power analysis of our study data revealed a substantially larger effect size (*f* = 1.899) with achieved power of 1.0, confirming that our study was adequately powered to detect significant differences between the intervention groups with our final sample of 34 participants. Considering a potential dropout rate of 10%, we recruited 34 participants who were randomly assigned to either the facial roller group (*n* = 17) or the gua sha group (*n* = 17) (Figure 1). Eligible participants met the following inclusion criteria: (1) no facial surgical procedures or invasive cosmetic treatments in the previous 12 months; (2) absence of professional facial treatments within the past 3 months; and (3) willingness to provide written informed consent. Participants were excluded if they: (1) had diagnosed facial skin conditions or dermatological disorders including eczema, severe acne, or rosacea; (2) reported previous allergic responses to massage tool materials such as jade or rose quartz; (3) were pregnant or lactating; (4) were using facial medications or topical treatments that might influence study outcomes; or (5) had facial piercings that could interfere with the application of the massage tools. Participants were also instructed to maintain their existing skincare routines without introducing new products or treatments during the 8‐week study period. Compliance was verified during weekly follow‐ups.

### 2.2 Instrumentations

Three measurement devices were used to assess facial contours, skin elasticity, and muscle tone parameters:

A three‐dimensional facial scanner (FastSCAN, Polhemus, USA) was used to capture detailed facial surface topography and anthropometric measurements. This non‐contact scanning system utilizes structured light technology to create high‐resolution 3D images of facial surfaces with an accuracy of ±0.5 mm. The scanner captures facial contours, volume, and surface area measurements, allowing for precise quantification of facial morphological changes. This system has demonstrated high reliability in previous studies for facial anthropometric measurements (ICC > 0.90) [6].

Skin elasticity was evaluated using the Cutometer MPA 580 (Courage + Khazaka Electronic GmbH, Köln, Germany), a non‐invasive device that measures the viscoelastic properties of skin [10]. The device creates negative pressure to draw the skin into a probe aperture and measures both the extent and rate of skin deformation. Key parameters measured include overall elasticity (R2) and elastic recovery (R7). The Cutometer has been extensively validated for skin elasticity measurements with excellent reliability (ICC = 0.86–0.92) [11].

Muscle tone and mechanical properties were assessed using the MyotonPRO (Myoton AS, Tallinn, Estonia), a portable device that measures tissue biomechanical properties through non‐invasive mechanical impulses [12]. The device quantifies muscle tone (Hz), dynamic stiffness (N/m), and elasticity (logarithmic decrement) of facial muscles. This technology has shown high reliability for facial muscle measurements and has been validated in previous studies examining facial muscle properties [13].

### 2.3 Facial Contour Measurements

Three‐dimensional facial scans were analyzed using Delta software (Farfield Technology, Christchurch, New Zealand) [6, 14]. To ensure measurement consistency between pre‐ and post‐intervention scans, data calibration was performed using three anatomical landmarks: the center of the Cupid's bow and bilateral sublobular points (the contact points under the ear lobules).

**FIGURE 2:**
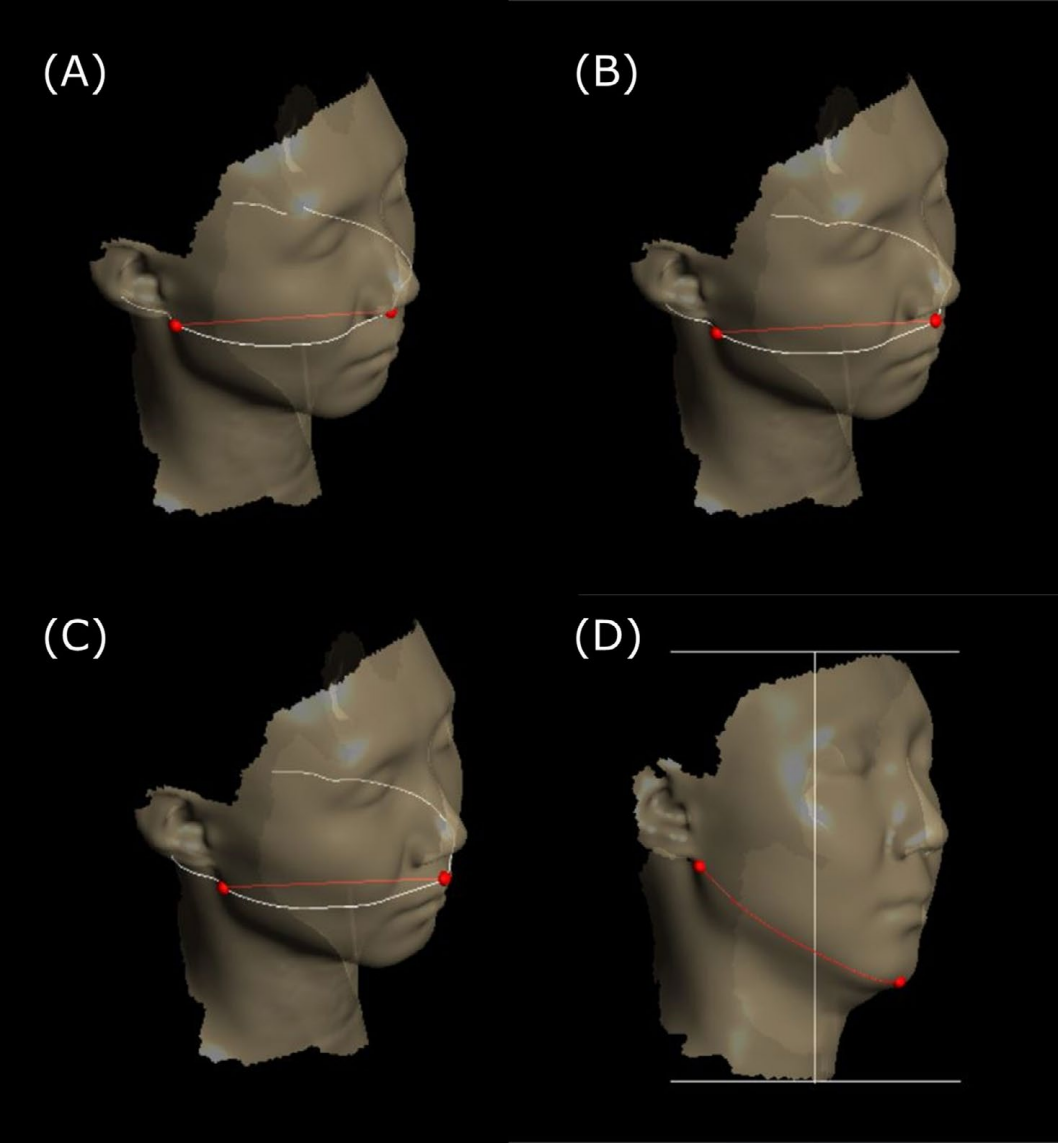
Measurement of Facial Contour: (A) measurement of subnasale‐to‐sublobular distance, (B) measurement of mid‐point distance, (C) measurement of labrale superius distance, (D) measurement of jawline surface distance.

Four specific surface distance measurements were obtained to assess changes in facial contours [14] (Figure 2). The mid‐facial distances included: (1) the subnasale‐to‐sublobular distance (SSD: from the subnasale to the bilateral sublobular points), (2) the mid‐point distance (MPD: from the midpoint between subnasale and labrale superius to the bilateral sublobular points), and (3) the labrale superius distance (LSD: from the labrale superius to the bilateral sublobular points). These three distances were measured bilaterally to evaluate improvements in the nasolabial folds. Additionally, (4) the jawline surface distance (JSD) was measured from a reference point (the intersection of the sagittal axis through the center of Cupid's bow and the chin in frontal view) to the contact spots on the ear lobe and chin bilaterally to assess improvements in jawline sagging.

All measurements were performed using the Contours mode in Delta software, with the sagittal axis aligned to the center of the Cupid's bow. For each measurement point, assessments were taken bilaterally, and the mean value of the right and left measurements was calculated. Each measurement was performed twice, and the average of these two measurements was used for statistical analysis. These standardized surface line measurements suggested high intra‐rater reliability (ICC = 0.894) [14].

### 2.4 Skin Elasticity Measurements

Skin elasticity was evaluated using the Cutometer MPA 580, which measures the viscoelastic properties of skin using a controlled negative pressure method [15]. Two key parameters were assessed: the gross elasticity (R2) and biological elasticity (R7). The R2 parameter, representing the ratio of immediate retraction to total distention (Ua/Uf), indicates the total recovery of skin deformation, where values closer to 1.0 represent greater skin elasticity. The R7 parameter, calculated as the ratio of immediate retraction to immediate distention (Ur/Uf), reflects the biological elasticity of the skin, with higher values indicating better elastic recovery [16].

Measurements were taken at two standardized facial points: the lateral canthus (outer corner of the eye) and the intersection point of the alar (nostril wing), following protocols established in previous facial elasticity studies. The device was configured with a probe aperture of 2 mm, negative pressure of 450 mbar, and on/off time of 2.0 s for each measurement cycle, settings that have been validated for facial skin measurements [11, 17]. Three consecutive measurements were performed at each point bilaterally, and the mean values of both sides were used for analysis. A one‐minute interval was maintained between measurements to allow for complete tissue recovery [17].

### 2.5 Muscle Tone Measurements

Muscle tone and mechanical properties were assessed using the MyotonPRO device, which applies a brief (15 ms) mechanical impulse to the skin surface overlying the muscle being tested [18]. The device measures two key parameters: oscillation frequency (Hz), which characterizes muscle tone (resting tension), and dynamic stiffness (N/m), which represents the muscle's resistance to contraction or shape deformation. Higher *F* values indicate increased muscle tone, while higher *S* values suggest greater muscle stiffness [13].

Measurements were taken at the mid‐point of the masseter muscle, identified by palpating the line between the mandibular angle and the midpoint of the zygomatic bone, following standardized protocols established in previous facial muscle studies [19]. This measurement location was chosen based on its reliability in assessing masseter muscle properties as demonstrated in previous research [20]. The device was positioned perpendicular to the muscle surface, and three consecutive measurements were performed on each side. The mean values of these three measurements were calculated for both the right and left masseter muscles, and the bilateral average was used for analysis. This measurement protocol has shown excellent reliability (ICC > 0.90) for masseter muscle assessment [20].

### 2.6 Procedure

**FIGURE 3:**
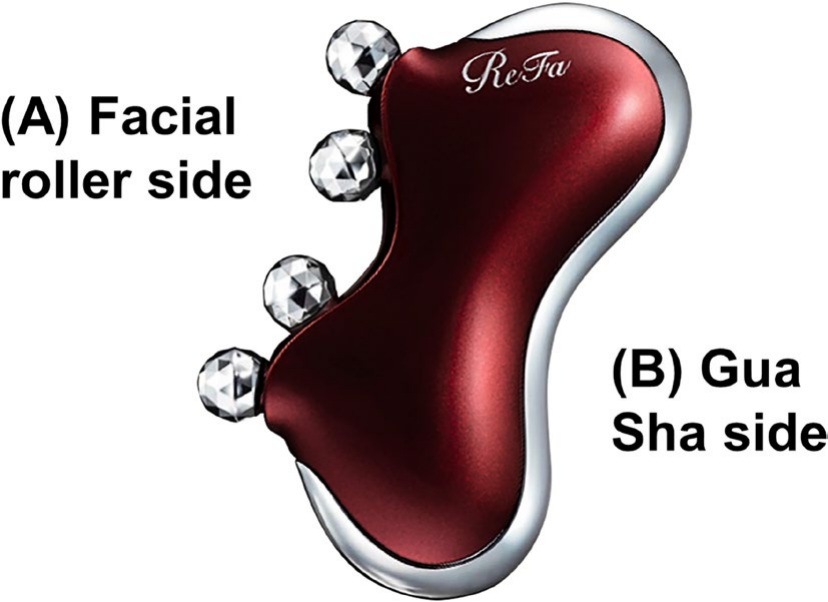
The dual‐function massage tool: (A) for facial rolling, (B) for gua sha massage.

**FIGURE 4:**
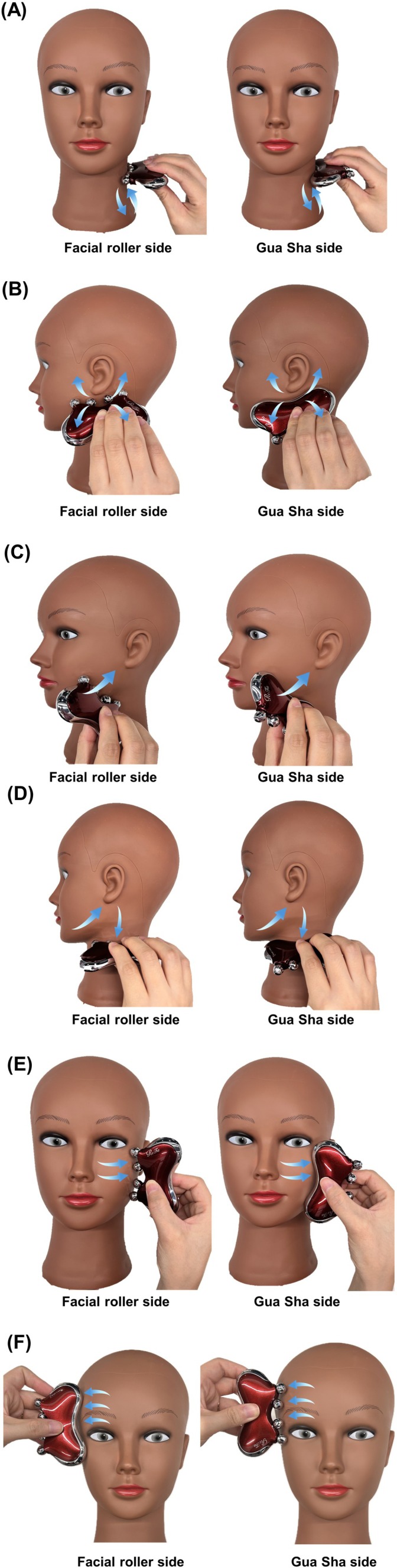
Step‐by‐step demonstration of the standardized massage protocol: (A) Preparatory strokes on neck and shoulders, (B) Relaxation of lymph nodes and jaw muscles, (C) Upward strokes from chin to ears, (D) Movements from chin to ears and down neck, (E) Massage from cheeks to temples, (F) Outward strokes along forehead and eyebrow line.

All facial massage interventions were performed using a dual‐function massage tool (ReFa CAXA M1, MTG Co. Ltd., Japan) [4], which features multi‐faceted facial rollers on one side, and a curved edge on the opposite side that serves as a gua sha tool (Figure 3). The device is made of ABS, polyacetal, stainless steel, silicone rubber, and chrome. The same tool was used for all participants, ensuring material consistency across both intervention groups. Group assignment was performed using a computer‐generated random allocation sequence obtained. To prevent detection bias, outcome examiners were blinded to group allocation throughout the study. The examiners responsible for conducting all measurements were not involved in participant recruitment, randomization, or intervention training. Participants were instructed not to disclose their assigned massage technique to the assessors during evaluation sessions. All measurements were conducted following identical protocols regardless of group assignment. This blinding procedure was maintained for both baseline and post‐intervention assessments. Following randomization, each group used only their designated side of the tool throughout the intervention period. During the initial assessment, all participants received comprehensive instruction and hands‐on training in their assigned massage technique. The training session included demonstration of proper tool use, pressure application, and movement patterns. Participants practiced the massage protocol under researcher supervision until they demonstrated competent execution of all steps, ensuring familiarization with the technique before beginning the intervention period. For the gua sha technique, participants were instructed to apply moderate pressure that produced visible skin displacement without bruising as the tool edge was drawn across the skin. This level of pressure was described as “firm enough to grip and move the superficial tissue, but not so firm as to cause discomfort or bruising.” For the facial roller technique, participants were instructed to apply consistent pressure sufficient to create visible compression of the skin beneath the roller without causing discomfort. This was described as “firm enough to feel the roller making an impression on the skin, but comfortable enough to maintain throughout the entire session.” The standardized massage protocol began with applying a small amount of pure facial oil to ensure smooth tool movement. The massage sequence consisted of: (1) preparatory strokes on the neck and shoulders, (2) relaxation of lymph nodes and jaw muscles near the ears, (3) upward strokes from the chin toward the ears, (4) movements from the chin to ears and down the neck, (5) massage from the cheeks to the temples, and (6) outward strokes along the forehead and eyebrow line (Figure 4). Each session concluded with gentle pressure on the temples and lasted 10 min.

Participants were instructed to perform this protocol five times per week for 8 weeks, resulting in a target compliance of 40 sessions over the intervention period. Weekly telephone follow‐ups were conducted to monitor adherence to the protocol. If participants reported fewer than five sessions in a week, they were encouraged to complete the missing sessions during the weekend to maintain the required frequency. All outcome measurements were conducted in the same sequence both at baseline and after the 8‐week intervention: facial three‐dimensional scanning was performed first, followed by skin elasticity measurements, and finally muscle tone assessment. This standardized measurement sequence was maintained to ensure consistency between pre‐ and post‐intervention evaluations.

### 2.7 Statistical Analysis

All statistical analyses were performed using SPSS version 18.0 (SPSS Inc., Chicago, IL, USA). The Kolmogorov–Smirnov test was used to assess the normality of data distribution for all variables. Demographic characteristics and baseline measurements between the facial roller and gua sha groups were compared using independent t‐tests. To evaluate the effects of the interventions, analysis of covariance (ANCOVA) was conducted with baseline values as covariates for each outcome measure (facial surface distances [SSD, MPD, LSD, JSD], skin elasticity parameters [R2, R7], and muscle tone measurements [*F*, *S*]). The between‐group factor was the intervention type (facial roller vs. gua sha), and the baseline measurements were used as covariates to control for initial differences. When significant main effects or interaction effects were detected, post hoc analyses were performed to identify specific timepoints differences. All data are presented as mean ± standard deviation, and statistical significance was set at *p* < 0.05.

## 3 Results

Thirty‐four participants were initially enrolled in the study, with one participant in the facial roller group dropping out due to lack of compliance for intervention sessions, resulting in 33 participants completing the study (gua sha group: *n* = 17; facial roller group: *n* = 16). No significant differences were observed in baseline demographic characteristics between the two groups (Table 1).

**TABLE 1:** Demographic characteristics of participants.

Variables	Gua sha group (*n* = 17)	Facial roller group (*n* = 16)	*p*
Age	45.59 ± 10.24	46.13 ± 10.50	0.883
Height	162.35 ± 6.53	158.06 ± 6.05	0.060
Weight	57.29 ± 7.14	55.94 ± 5.00	0.534
BMI	21.70 ± 2.05	22.53 ± 2.98	0.358

Abbreviation: BMI, body mass index.

Both groups showed significant improvements in facial contour measurements over time (*p* < 0.001) (Table 2). All four facial surface distances (SSD, MPD, LSD, and JSD) decreased significantly from baseline in both groups, with no significant between‐group differences. The mean reductions in facial surface distances ranged from 2.23 to 2.40 mm in the gua sha group and from 2.75 to 3.26 mm in the facial roller group. These reductions exceed the threshold of 2.0 mm that has been associated with visually perceptible changes in facial contours [6] and are comparable to changes reported in previous studies where clinical improvements were observed. The magnitude of these changes is consistent with what would be expected to produce noticeable improvements in nasolabial fold appearance and jawline definition.

**TABLE 2:** Comparison of facial contours between the two groups before and after interventions.

Variables	Group	Pre	Post	Change mean (95% CI)	*p*	*p* (between group)	*p* (within time)
SSD	Gua sha group	131.18 ± 5.15	128.79 ± 5.44	2.40 (1.61 −3.18)	0.000	0.352	0.000
	Facial roller group	129.00 ± 5.34	126.21 ± 4.69	2.78 (1.81 −3.75)	0.000		
MPD	Gua sha group	129.87 ± 4.87	127.48 ± 5.40	2.39 (1.42 −3.37)	0.000	0.152	0.000
	Facial roller group	128.31 ± 5.29	125.06 ± 4.87	3.26 (2.35 −4.16)	0.000		
LSD	Gua sha group	129.15 ± 4.77	126.83 ± 5.31	2.32 (1.56 −3.07)	0.000	0.132	0.000
	Facial roller group	127.58 ± 5.26	124.47 ± 4.73	3.11 (2.22 −4.01)	0.000		
JSD	Gua sha group	125.32 ± 5.11	123.09 ± 5.50	2.23 (1.48 −2.99)	0.000	0.263	0.000
	Facial roller group	126.01 ± 5.57	123.26 ± 5.99	2.75 (2.07 −3.42)	0.000		

Abbreviations: JSD, jawline surface distance (measurement from a reference point to the contact spots on the ear lobe and chin bilaterally); LSD, labrale superius distance (measurement from the labrale superius to the bilateral sublobular points); MPD, mid‐point distance (measurement from the midpoint between subnasale and labrale superius to the bilateral sublobular points); SSD, subnasale‐to‐sublobular distance (measurement from the subnasale to the bilateral sublobular points).

**FIGURE 5:**
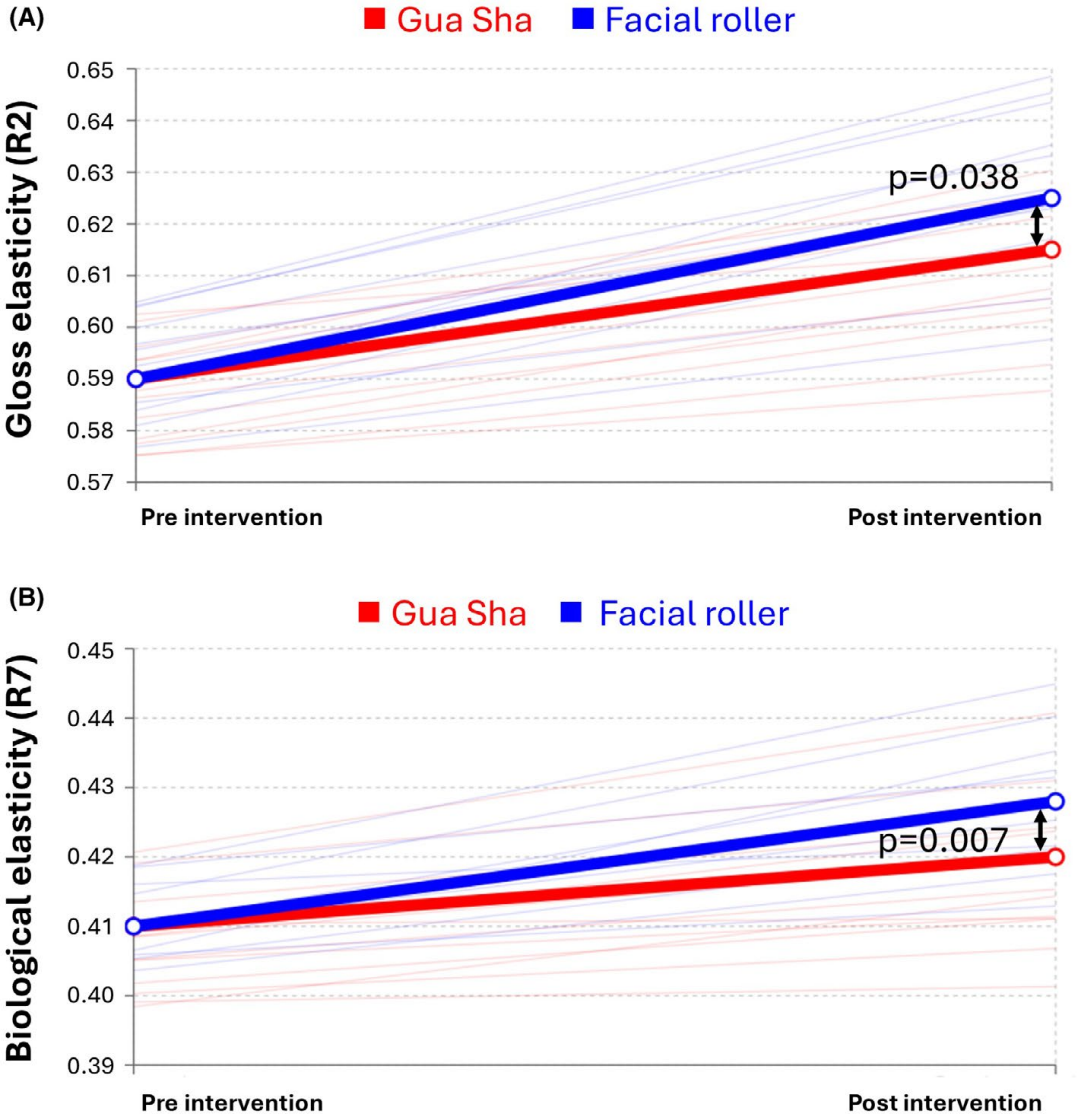
Comparisons of skin elasticity between Gua Sha and facial roller massage: Values represent adjusted post‐intervention measurements with baseline values as covariates in ANCOVA analysis: (A) gross elasticity (R2), higher values indicate better overall elastic recovery; (B) biological elasticity (R7), higher values indicate improved elastic recovery relative to distention.

For skin elasticity parameters, the facial roller group showed more pronounced improvements compared to the gua sha group (Table 3 and Figure 5). Gross elasticity (R2) increased significantly in the facial roller group (−0.05; 95% CI: −0.06–0.03; *p* < 0.001) but not in the gua sha group (−0.02; 95% CI: −0.04–0.01; *p* = 0.129), with significant between‐group differences (*p* = 0.038). Similarly, biological elasticity (R7) improved significantly in the facial roller group (−0.03; 95% CI: −0.04–0.02; *p* < 0.001) but not in the gua sha group (0.00; 95% CI: −0.02–0.01; *p* = 0.675), with significant between‐group differences (*p* = 0.007).

**TABLE 3:** Comparison of muscle tone and skin elasticity between the two groups before and after interventions.

Variables	Group	Pre	Post	Change mean (95% CI)	*p*	*p* (between group)	*p* (within time)
*F* (Hz)	Gua sha group	19.96 ± 2.72	17.94 ± 3.07	2.02 (1.09 to 2.96)	0.000	0.005	0.007
	Facial roller group	20.06 ± 3.55	20.10 ± 3.21	−0.05 (−1.24 to 1.15)	0.937		
*S* (N/m)	Gua sha group	424.85 ± 70.19	368.40 ± 75.82	56.46 (23.16 to 89.75)	0.002	0.030	0.002
	Facial roller group	448.53 ± 102.15	431.23 ± 82.19	17.30 (−22.56 to 57.15)	0.370		
R2 (Ua/Uf)	Gua sha group	0.60 ± 0.05	0.62 ± 0.05	−0.02 (−0.04 to 0.01)	0.129	0.038	0.000
	Facial roller group	0.58 ± 0.04	0.63 ± 0.05	−0.05 (−0.06 to −0.03)	0.000		
R7 (Ur/Uf)	Gua sha group	0.42 ± 0.05	0.42 ± 0.05	0.00 (−0.02 to 0.01)	0.675	0.007	0.001
	Facial roller group	0.40 ± 0.03	0.43 ± 0.04	−0.03 (−0.04 to −0.02)	0.000		

Abbreviations: *F*, oscillation frequency (measured in Hz, characterizes muscle tone or resting tension); R2, gross elasticity (ratio of immediate retraction to total distention, Ua/Uf); R7, biological elasticity (ratio of immediate retraction to immediate distention, Ur/Uf); *S*, dynamic stiffness (measured in N/m, represents the muscle's resistance to contraction).

**FIGURE 6:**
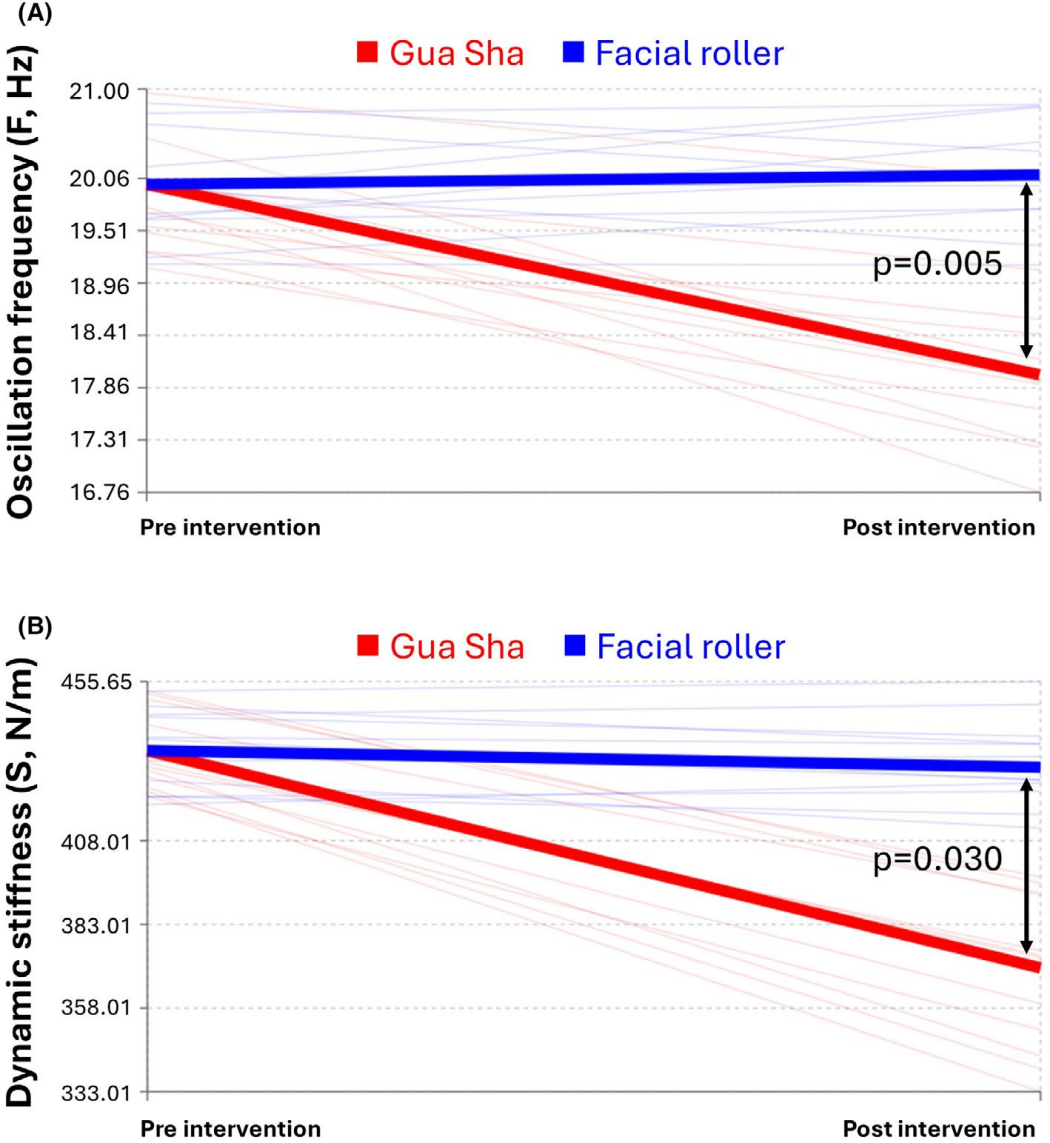
Comparisons of muscle tone between Gua Sha and facial roller massage: Values represent adjusted post‐intervention measurements with baseline values as covariates in ANCOVA analysis: (A) oscillation frequency (*F*, Hz), where lower values indicate reduced muscle tone; (B) dynamic stiffness (*S*, N/m), where lower values indicate reduced resistance to deformation.

Regarding muscle tone parameters, significant between‐group differences were observed (Table 3 and Figure 6). The gua sha group demonstrated significant reductions in both oscillation frequency (2.02 Hz; 95% CI: 1.09–2.96; *p* < 0.001) and dynamic stiffness (56.46 N/m; 95% CI: 23.16–89.75; *p* = 0.002), while the facial roller group showed no significant changes. The between‐group differences were significant for both *F* (*p* = 0.005) and *S* (*p* = 0.030).

## 4 Discussion

The present study compared the effects of facial roller and gua sha massages on facial contours, muscle tone, and skin elasticity. A key finding was that these similar contouring outcomes were achieved through distinctly different physiological mechanisms: the gua sha group primarily through changes in muscle properties and deep tissue mobilization, and the facial roller group predominantly through enhanced skin elasticity. This distinction in mechanisms, despite similar contouring outcomes, represents a novel and important finding in facial aesthetic research.

The observed improvements in facial surface distances were both statistically significant and clinically relevant across both intervention groups. The gua sha group showed reductions ranging from 2.23 to 2.40 mm, while the facial roller group demonstrated changes of 2.75 to 3.26 mm, both comparable to the findings of van de Velde et al. [6], who reported average reductions of 2.1–3.0 mm following manual lymphatic drainage. In the gua sha group, these changes were accompanied by significant reductions in muscle tone parameters, with oscillation frequency decreasing by 2.02 Hz (95% CI: 1.09–2.96) and dynamic stiffness reducing by 56.46 N/m (95% CI: 23.16–89.75). These findings align closely with previous research by Cheatham et al. [8], who demonstrated that IASTM techniques reduced tissue resistance by 31%–51% through fascial mobilization. The consistency between our findings and previous research strengthens the validity of our results and suggests a reliable therapeutic effect. Moreover, the magnitude of change in our study exceeded some previously reported outcomes, possibly due to our longer intervention period and standardized application protocol.

The facial roller group demonstrated particularly noteworthy improvements in skin elasticity parameters, with significant increases in both gross elasticity (R2) and biological elasticity (R7). These improvements, showing increases of 8.6% and 7.5% respectively, reflect the fundamental mechanical properties of skin as described by Biggs et al. [5], who emphasized how mechanical forces influence tissue architecture and stability. The enhanced effect observed in our study might be attributed to several factors, including our longer intervention duration of 8 weeks compared to previous mechanical intervention studies that typically lasted 4 weeks [4]. Mechanical forces in skin tissues have been shown to play crucial roles in maintaining tissue architecture and function through mechanotransduction pathways [5, 21], which may explain the cumulative benefits we observed with regular facial roller application [21]. The consistency of application and our standardized protocol may have also contributed to these superior outcomes by ensuring optimal mechanical stimulation of the superficial tissue layers [21, 22]. The absence of significant changes in muscle tone parameters in this group, coupled with the significant improvements in skin elasticity, further supports the specificity of the roller's effects on superficial tissue structures, aligning with previous findings on compartment‐specific structural specialization in skin responses to mechanical forces [5, 23, 24].

Several distinct physiological mechanisms likely account for these differential outcomes between the two interventions. Gua sha's scraping motion generates mechanical stress that directly impacts muscle and fascial tissues through compartment‐specific structural specialization [5, 7]. The sustained pressure and scraping motion characteristic of gua sha technique activates mechanoreceptors in muscle tissue, leading to reduced tone through neuromuscular mechanisms [7]. This deeper tissue engagement promotes increased blood flow and tissue oxygenation, with effects lasting up to 25 min post‐treatment [4]. In contrast, roller massage creates rhythmic compression patterns primarily affecting superficial tissues, preferentially stimulating fibroblast activity in the dermis and enhancing superficial circulation for 10–15 min post‐application [4]. The mechanical stimulation provided by facial rollers activates mechanoreceptors in the dermis, triggering mechanotransduction pathways that regulate collagen and elastin production in fibroblasts. This dermal remodeling appears to primarily influence the skin's elastic properties rather than underlying muscle tone, explaining our observation of improved skin elasticity parameters without significant changes in muscle properties in the roller group. The different circulatory effects between these techniques may explain the varying impacts on tissue properties and the subsequent improvements in facial contours through distinct pathways, reflecting the fundamental mechanical properties and organization of different skin layers [5].

When comparing facial roller and gua sha massage with energy‐based devices (EBDs) and high‐intensity focused ultrasound (HIFU) techniques for skin tightening, several key differences emerge. While our study found that facial roller primarily influences skin elasticity parameters and gua sha affects muscle tone properties, EBDs operate through different mechanisms. EBDs convert various forms of energy into heat, creating thermal coagulation points at specific tissue depths, targeting the SMAS layer at temperatures exceeding 60°C [25]. These thermal effects induce immediate collagen contraction followed by neocollagenesis over 3–6 months post‐treatment. Microfocused ultrasound with visualization (MFU‐V) can produce small (< 1 mm^3^) thermal coagulation points at depths of 1.5–4.5 mm [26], with clinical studies showing brow lifts of 0.47–1.7 mm and submental area reductions of 26–45 mm^2^ [27]. EBDs typically require 90–180 days to achieve maximum clinical benefit, with global aesthetic improvement scales showing improvement in 92% of patients at day 90 post‐treatment [27]. Unlike our manual techniques, which produce immediate effects on muscle tone and skin elasticity with negligible adverse effects, energy‐based methods carry risks including burns, dysesthesia, bruising, and rarely lipoatrophy [25]. The non‐invasive nature of facial roller and gua sha massage positions these traditional methods as complementary approaches to more technically sophisticated EBDs, particularly for patients seeking immediate improvements without downtime or those requiring maintenance between more intensive treatments. From a clinical decision‐making perspective, these differences suggest complementary roles rather than competing alternatives. Manual techniques like facial roller and gua sha may be more appropriate for maintenance therapy, patients seeking gradual improvements without downtime, those with contraindications to EBDs, or as preparatory interventions before more intensive procedures. EBDs may be preferred for patients requiring more dramatic or deeper tissue effects, those with significant skin laxity, or cases where precise targeting of specific anatomical planes is necessary. The non‐invasive nature and favorable safety profile of facial roller and gua sha massage position these traditional methods as valuable components in comprehensive facial rejuvenation protocols, either as standalone treatments or complementary to more technically sophisticated approaches.

These findings have important and direct clinical implications for practitioners in facial aesthetic treatments. The choice between gua sha and facial roller techniques can now be more precisely tailored to individual patient needs and treatment goals. For patients presenting with increased muscle tension or deep tissue restrictions contributing to facial contours, gua sha massage would be the more appropriate choice due to its demonstrated effects on muscle properties and deep tissue structures. This recommendation is particularly relevant for cases where facial appearance is affected by muscular factors such as masseteric hypertrophy or increased muscle tone. Conversely, patients primarily concerned with skin elasticity and surface tissue properties would likely benefit more from facial roller treatments, as evidenced by the significant improvements in elasticity parameters observed in our study. The specific effects of each technique also suggest potential benefits in combining both approaches for comprehensive facial rejuvenation protocols.

Several limitations of the current study should be carefully considered when interpreting and applying these findings. First, while the eight‐week intervention period was sufficient to demonstrate significant changes, it may not fully reflect the long‐term sustainability of these improvements or potential cumulative effects over extended periods. Future research should consider longitudinal follow‐up studies to assess the maintenance of these improvements. Second, although our sample size was statistically adequate for detecting significant effects, larger‐scale studies would enhance the generalizability of these findings across diverse populations. Additionally, our study focused on healthy adults within a specific age range, and the results may vary in different age groups or in individuals with specific skin conditions or facial muscle disorders. Third, the standardized application protocol, while ensuring consistency, may not reflect the variations in technique that occur in real‐world settings. Therefore, future studies should investigate the effects of these interventions under more diverse practical conditions. Fourth, the present study is the absence of standardized clinical photographs to visually document the changes in facial contours. While our objective measurements using three‐dimensional scanning provided precise quantitative data, the inclusion of before‐and‐after clinical images would have offered readers a more intuitive visual representation of the treatment outcomes. Future studies should incorporate standardized clinical photography with appropriate privacy protections and ethical approvals to better illustrate the visual effects of these interventions. Finally, the current study is the absence of a third intervention group that combined both facial roller and gua sha techniques. Given our finding that these techniques work through different physiological mechanisms, investigating their potential synergistic effects when used in combination would be valuable for comprehensive facial rejuvenation protocols. Future research should examine whether combining both techniques could potentially enhance outcomes beyond what either technique achieves individually, and determine optimal sequencing and frequency when using both modalities together.

## 5 Conclusions

This study demonstrates that both facial roller and gua sha massage techniques effectively improve facial contours through distinct physiological mechanisms. While gua sha massage primarily influences muscle tone and deeper tissue properties, facial roller treatment predominantly affects skin elasticity and superficial tissue characteristics. Our results reveal that similar aesthetic outcomes can be achieved through different pathways, allowing practitioners to select techniques based on specific treatment targets. These findings have direct clinical implications for treatment selection in aesthetic practice. Practitioners should consider recommending gua sha massage for patients presenting with increased facial muscle tension, masseteric hypertrophy, or conditions where reduced muscle tone would be beneficial. Conversely, facial roller massage would be more appropriate for patients primarily concerned with skin elasticity, early signs of skin laxity, or as part of a preventative regimen focused on maintaining skin firmness. For comprehensive facial rejuvenation, clinicians might consider the sequential application of both techniques, with gua sha addressing deeper tissue restrictions followed by facial roller to enhance superficial skin properties. Future research should investigate the long‐term sustainability of these improvements and explore the potential synergistic effects of combining both techniques in integrated treatment protocols.

## Author Contributions

Ui‐jae Hwang and Sun‐hee Ahn contributed to conceptualization, methodology, writing – original draft, and visualization. Ui‐jae Hwang and Hyo Sun Han contributed to supervision and project administration. Jun‐Hee Kim and A‐Hyun Hwang contributed to data curation, validation, and software. Jun‐Hee Kim, Hyun‐Joo Lee, Yu‐Rin Jeon, A‐Hyun Hwang, and Hyun Hwa Lee contributed to data curation and formal analysis.

## Ethics Statement

The present study conformed to the ethical guidelines of the 1975 Declarations of Helsinki. This study was approved by the Yonsei University Mirae Campus Institutional Review Board. Informed consent for publication of the images was obtained from the patient.

## Conflicts of Interest

The authors declare no conflicts of interest.

## Data Availability

The datasets analyzed during the current study are available from the corresponding author upon reasonable request.
